# Associations of physical activity and egg intake with hypertension among Chinese middle-aged and older population

**DOI:** 10.1038/s41598-019-43966-1

**Published:** 2019-05-22

**Authors:** Haizhen He, Ting Zhang, Jing Zhou, Zhu Zhu, Xiaona Na, Guirong Zhou, Guoliang Zhuang, Aiping Liu

**Affiliations:** 1 Department of Social Medicine and Health Education, School of Public Health, Peking University, Beijing, China; 2 Disease Prevention and Control Center of Mentougou, Beijing, China

**Keywords:** Risk factors, Lifestyle modification

## Abstract

To explore the independent and interaction associations of physical activity (PA) and egg intake with hypertension. A cross-sectional study of 2189 individuals (aged ≥50 years) selected using multi-stage stratified random sampling was conducted in Mentougou of Beijing, China. Data of PA and egg intake were obtained from questionnaire survey, and blood pressure from physical examination. Individuals were divided into four groups by the level of PA: low, medium, high level 1 and high level 2; and were also divided into three groups by daily amount of egg intake: <1 egg/day, 1 egg/day and >1 egg/day. Self-reported hypertension was defined if individual had a self-reported diagnosis of hypertension or use of antihypertensive medication; examined abnormal blood pressure was defined if individual didn’t belong to self-reported hypertension and mean blood pressure was above 130/80 mmHg measured during this examination. After adjusting demographic characteristics, health behavior, BMI, and family history of CVD, compared with medium level PA group, high level 2 group was associated with higher self-reported rate (OR: 1.5, 95% CI: 1.18–2.01) and examined abnormal rate (OR: 1.8, 95% CI: 1.21–2.20). The self-reported rate and examined abnormal rate in <1 egg/day group were both higher than 1 egg/day group (OR: 1.4, 95% CI: 1.06–1.91; OR: 1.4, 95% CI: 1.08–1.92). Compared with medium level PA and 1 egg/day group, high level 2 and >1 egg/day group with higher examined abnormal rate (OR: 2.3, 95% CI: 1.28–4.01), high level 2 and <1 egg/day group with higher self-reported rate and examined abnormal rate (OR: 2.5, 95% CI: 1.10–3.82; OR: 2.9, 95% CI: 1.54–4.96). In conclusion, excessive high level PA and inadequate egg intake were associated with hypertension, respectively, and the association further increased when both existed at the same time. Besides, moderate PA combined with reasonable egg intake was associated with the low examination rate of hypertension.

## Introduction

In recent years, cardiovascular disease is still the main cause of death and disability all over the world, including China^1^. Hypertension is widely acknowledged as the most common cardiovascular disorder, affecting one in four adults worldwide^2^. The prevalence of hypertension is increasing globally, and it is estimated to rise to 30% by the year 2025^2^. In China, the patient number with hypertension was up to 270 million based on the 2010 sixth national population census data, and mainly concentrated in the middle-aged and old people^3,4^.

Despite of its high prevalence, hypertension has a low rate of successful treatment, so that more and better primary prevention through lifestyle modifications may be a major public health priority. A large number of studies have shown that physical activity, diet and other behavioral factors play an important role in the occurrence and development of hypertension, as do non-confounded factors such as age and sex^5^. Moderate level PA are conducive to the prevention and treatment of hypertension^6–8^. Eggs are a main source of dietary cholesterol, but they also contain all kinds of vitamins, high-quality protein, and other nutrients, so previous studies have no inconsistent conclusion about the association between egg intake and cardiovascular disease, and most of them found insignificant associations^9^. However, a recent prospective study^1^ reported that compared with non-consumers, daily egg consumption (usual amount 0.76 egg/day) was associated with lower risk of CVD (OR 0.89).

Numerous studies have shown that increasing PA requires protein supplementation^10,11^. In the process of exercise, protein is needed to build muscles and provide energy, and if protein supplement is not available, it may cause harm to health^10^. Figueroa *et al*.^11^ showed that combining exercise with milk can reduce systolic blood pressure. Feng *et al*.^5^ found that the risk of hypertension was significantly reduced when the combination of dietary pattern (dominated by high-protein foods) and PA. We all know that eggs are the main source of high-quality protein and biological value is particularly significant, due to the amino acid composition is similar with human body. In the traditional dietary pattern of China, eggs are one of the main sources of food protein, and the consumption is huge^12^. However, most previous studies focused on the single association of PA and egg intake with hypertension, and there were few studies on the interaction association of both, especially in China. We conducted a cross-sectional study to explore the single and interaction associations of PA and egg intake with hypertension in Chinese middle-aged and older population, which may be a significant supplementary on studies of hypertension and help provide reference for more effective interventions.

## Results

The average age of 2189 participants was 60.1 ± 7.3 years. There were 884 men (40.4%) with an average age of 59.9 ± 7.5 years; 1305 women (59.6%) and an average age of 60.2 ± 7.3 years.

Among 2026 participants, 62 (3.1%), 829 (40.9%), 669 (33.0%) and 466 (23.0%) were in the low, medium, high level 1 and high level 2 PA group, respectively. The distribution of PA was different among different gender, occupation, smoking, drinking, cereal and tuber intake, livestock and poultry meat intake population (all P < 0.05).

Of the 2136 participants, <1 egg/day, 1 egg/day and >1 egg/day group were 680 (31.8%), 780 (36.5%) and 676 (31.6%), respectively. The distribution of egg intake was statistically significant (all P < 0.05) in different occupation, smoking, cereal and tuber, livestock and poultry meat and milk population (Table 1).

**Table 1:** Distribution of PA and egg intake in different characteristic population (n/%).

Item	PA							Egg intake					
	n	High level 2	High level 1	Medium level	Low level	χ^2^	P	n	>1 egg/day	1 egg/day	<1 egg/day	χ^2^	P
**Gender**													
men	831	146 (17.6)	257 (30.9)	387 (46.6)	41 (4.9)	47.1	0.000	861	255 (29.6)	311 (36.1)	295 (34.3)	4.6	0.100
women	1195	320 (26.8)	412 (34.5)	442 (37.0)	21 (1.8)			1275	421 (33.0)	469 (36.8)	385 (30.2)		
**Age**(**years**)													
≥65	534	115 (21.5)	173 (32.4)	233 (43.6)	13 (2.4)	2.9	0.392		194 (35.3)	182 (33.1)	174 (31.6)	5.5	0.065
<65	1492	351 (23.5)	496 (33.2)	596 (39.9)	49 (3.3)				482 (30.4)	598 (37.7)	506 (31.9)		
**Occupation**													
productive labor	523	139 (26.6)	185 (35.4)	190 (36.6)	9 (1.7)	77.8	0.000	558	217 (38.9)	152 (27.2)	189 (33.9)	52.8	0.000
services	158	23 (14.6)	50 (31.6)	75 (47.5)	10 (6.3)			167	46 (27.5)	68 (40.7)	53 (31.7)		
retired	637	156 (24.5)	196 (30.8)	270 (42.4)	15 (2.4)			678	203 (29.9)	280 (41.3)	195 (28.8)		
others	696	144 (20.7)	234 (33.6)	292 (42.0)	26 (3.7)			719	203 (28.2)	275 (38.2)	241 (33.5)		
**personal annual income**(**yuan**)													
≥2800	1746	400 (22.9)	569(32.6)	724(41.5)	53(3.0)	1.7	0.634		595 (32.4)	665 (36.2)	578 (31.4)	3.2	0.200
<2800	280	66 (23.6)	100 (35.7)	105 (37.5)	9 (3.2)				81 (27.2)	115 (38.6)	102 (34.2)		
**smoking** (**cigarette/day**)													
<1	1566	366 (23.4)	541 (34.5)	618 (39.5)	41 (2.6)	18.4	0.005	1630	544 (32.9)	604 (36.5)	505 (30.6)	19.8	0.001
≥1	408	84 (20.6)	112 (27.5)	191 (46.8)	21 (5.1)			425	106 (24.9)	158 (37.2)	161 (37.9)		
**drinking** (**time/month**)													
<1	1541	374 (24.3)	522 (33.9)	607 (39.4)	38 (2.5)	17.4	0.001	1617	533 (33.0)	574 (35.5)	510 (31.5)	5.8	0.055
≥1	483	92 (19.0)	145 (30.0)	222 (46.0)	24 (5.0)			517	142 (27.5)	205 (39.7)	170 (32.9)		
**BMI**													
<24	621	140 (22.5)	199 (32.0)	264 (42.5)	18 (2.9)	5.1	0.531	654	208 (31.8)	239 (36.5)	207 (31.7)	4.6	0.328
24∼	937	219 (23.4)	310 (33.1)	385 (41.1)	23 (2.5)			987	325 (32.9)	365 (37.0)	297 (30.1)		
28∼	465	107 (23.0)	159 (34.2)	179 (38.5)	20 (4.3)			489	142 (29.0)	174 (35.6)	173 (35.4)		
**cereal and tuber** (**g/day**)													
<250	350	65 (18.6)	111 (31.7)	157 (44.9)	17 (4.9)	19.7	0.003	357	84 (23.5)	134 (37.5)	139 (38.9)	19.1	0.001
250∼	938	246 (26.2)	298 (31.8)	366 (39.0)	28 (3.0)			1003	324 (32.3)	388 (38.7)	291 (29.0)		
400∼	678	135 (19.9)	246 (36.3)	281 (41.4)	16 (2.4)			721	244 (33.8)	247 (34.3)	230 (31.9)		
**livestock and poultry meat**(**g/day**)													
<40	819	180 (22.0)	257 (31.4)	356 (43.5)	26 (3.2)	7.4	0.022	854	228 (26.7)	291 (34.1)	335 (39.2)	42	0.000
40∼	521	110 (21.1)	183 (35.1)	212 (40.7)	16 (3.1)			566	183 (32.3)	214 (37.8)	169 (29.9)		
75∼	673	173 (25.7)	224 (33.3)	256 (38.0)	20 (3.0)			713	263 (36.9)	274 (38.4)	176 (24.7)		
**milk** (**ml/day**)													
<300	1875	440 (23.5)	613 (32.7)	763 (40.7)	59 (3.1)	2.4	0.491	1993	614 (30.8)	727 (36.5)	652 (32.7)	15	0.001
≥300	119	21 (17.6)	42 (35.3)	53 (44.5)	3 (2.5)			125	55 (44.0)	48 (38.4)	22 (17.6)		
**total**	2026	466 (23.0)	669 (33.0)	829 (40.9)	62 (3.1)			2136	676 (31.6)	780 (36.5)	680 (31.8)		

In the total, men and women participants, the self-reported rate was 36.4%, 36.2% and 36.6% respectively, and the examined abnormal rate were 37.9%, 43.1% and 34.4%, respectively. Men was with higher the examined abnormal rate than women (P < 0.05).

Compared with the medium level PA group, the self-reported rate in the high level 1 and the high level 2 PA group all decreased, and the examined abnormal rate in the high level 1 PA group increased slightly, but there was no statistically significant. The examined abnormal rate in the high level 2 PA group was higher (P < 0.01) than the medium level PA group, and was also higher than the high level 1 PA group (P < 0.05). The multiple logistic regression showed the high level 2 group was associated with higher self-reported rate (OR: 1.5, 95% CI: 1.18–2.01) and examined abnormal rate (OR: 1.8, 95% CI: 1.21–2.20) compared with the medium level PA group in model 3. The self-reported rate and examined abnormal rate were all increased in the low level PA group than the medium level PA group, but all the difference was not statistically significant (Table 2).

**Table 2:** Associations of PA and egg intake with hypertension.

classification	N	Self-reported hypertension					Examined abnormal blood pressure				
		n/%	Unadjusted OR(CI)	Adjusted OR(CI)			n/%	Unadjusted OR(CI)	Adjusted OR(CI)		
				Model 1	Model 2	Model 3			Model 1	Model 2	Model 3
**PA**											
Medium level	829	315 (38.0)	1.0	1.0	1.0	1.0	290 (35.0)	1.0	1.0	1.0	1.0
Low level	62	26 (41.9)	1.7 (0.81–3.47)	1.5 (0.74–3.36)	1.5 (0.73–3.35)	1.3 (0.75–3.25)	25 (40.3)	1.7 (0.84–3.64)	1.6 (0.78–3.62)	1.7 (0.83–3.87)	1.1 (0.81–3.68)
High level 1	669	237 (35.4)	0.9 (0.73–1.23)	0.9 (0.76–1.30)	0.9 (0.73–1.28)	0.9 (0.76–1.18)	255 (38.1)	1.1 (0.85–1.44)	1.1 (0.86–1.47)	1.0 (0.81–1.41)	1.1 (0.80–1.39)
High level 2	466	161 (34.5)	1.1 (0.84–1.55)	1.2 (0.87–1.63)	1.2 (0.89–1.73)	1.5 (1.18–2.01)^a^	205 (44.0)^a,b^	1.6 (1.17–2.12)^a^	1.6 (1.22–2.25)^a^	1.7 (1.24–2.36)^a^	1.8 (1.21–2.20)^a^
**Egg intake**											
1 egg/day	780	276 (35.4)	1.0	1.0	1.0	1.0	277 (35.5)	1.0	1.0	1.0	1.0
>1 egg/day	676	235 (34.8)	1.1 (0.84–1.42)	1.0 (0.82–1.42)	1.1 (0.79–1.39)	1.1 (0.75–1.29)	265 (39.2)	1.2 (0.95–1.59)	1.2 (0.92–1.57)	1.2 (0.91–1.60)	1.1 (0.89–1.59)
<1 egg/day	680	257 (37.8)^c^	1.4 (1.10–1.88)^c^	1.4 (1.06–1.84)^c^	1.4 (1.08–1.93)^c^	1.4 (1.06–1.91)^c^	276 (40.6)^c^	1.5 (1.17–2.00)^c^	1.4 (1.07–1.85)^c^	1.5 (1.09–1.95)^c^	1.4 (1.08–1.92)^c^

Normal blood pressure group was the reference; compared with the medium level PA group, ^a^P < 0.05; compared with the high level 1 PA group, ^b^P < 0.05; compared with 1 egg/day group, ^c^P < 0.05. Model 1 adjusted gender, age, occupation and personal annual income; model 2 adjusted smoking, drinking, cereal and tuber, livestock and poultry meat and milk based on model 1; model 3 adjusted BMI and family history of CVD based on model 2.

Compared with the 1 egg/day group, the self-reported rate and examined abnormal rate all increased (OR: 1.4, 95% CI: 1.06–1.91; OR: 1.4, 95% CI: 1.08–1.92) in the <1 egg/day group in model 3; the self-reported rate decreased and the examined abnormal rate increased in the >1 egg/day group, and all the difference was not statistically significant (Table 2).

Compared with the medium level PA and 1 egg/day group, the high level 2 PA and >1 egg/day group with higher examined abnormal rate (OR: 2.3, 95% CI: 1.28–4.01), and the high level 2 PA and <1 egg/day group with higher self-reported rate and examined abnormal rate (OR: 2.5, 95% CI: 1.10–3.82; OR: 2.9, 95% CI: 1.54–4.96) in model 3 (Table 3). For the low level PA group, combining <1 egg/day and >1 egg/day group due to the small sample size, and self-reported rate and examined abnormal rate were all increased than 1 egg/day group, but all the difference was not statistically significant.

**Table 3:** The interaction association of PA and egg intake with hypertension.

PA	N	Self-reported hypertension					Examined abnormal blood pressure				
		n/%	Unadjusted OR(CI)	Adjusted OR(CI)			n/%	Unadjusted OR(CI)	Adjusted OR(CI)		
				Model 1	Model 2	Model 3			Model 1	Model 2	Model 3
**Medium level**											
Egg intake											
1 egg/day	302	104 (34.4)	1.0	1.0	1.0	1.0	105 (34.8)	1.0	1.0	1.0	1.0
>1 egg/day	218	85 (39.0)	1.3 (0.86–2.06)	1.3 (0.84–2.08)	1.3 (0.84–2.13)	1.5 (0.72–3.53)	76 (34.9)	1.1 (0.75–1.83)	1.1 (0.73–1.82)	1.2 (0.77–1.96)	1.1 (0.68–1.85)
<1 egg/day	288	109 (37.8)	1.3 (0.88–2.00)	1.3 (0.88–2.03)	1.4 (0.96–2.31)	1.3 (0.68–2.23)	106 (36.8)	1.2 (0.85–1.93)	1.1 (0.78–1.80)	1.3 (0.86–2.09)	1.2 (0.84–2.10)
**Low level**											
Egg intake											
1 egg/day	15	8 (53.3)	1.7 (0.52–6.13)	1.8 (0.50–6.42)	1.6 (0.44–6.02)	1.3 (0.52–5.13)	3 (20.0)	0.6 (0.14–3.04)	0.6 (0.14–3.22)	0.7 (0.16–3.73)	0.5 (0.18–3.82)
>1 egg/day	18	8 (44.4)	2.3 (0.61–9.25)	2.0 (0.49–8.28)	2.0 (0.50–8.46)	1.6 (0.92–7.03)	7 (38.9)	2.0 (0.51–8.22)	1.7 (0.42–7.56)	1.9 (0.47–8.49)	0.8 (0.56–8.25)
<1 egg/day	29	10 (34.5)	2.2 (0.67–7.36)	2.1 (0.64–7.41)	2.3 (0.69–8.16)	2.1 (0.56–8.32)	15 (51.7)	3.3 (1.06–10.36)^a^	3.0 (0.93–10.07)	3.5 (1.07–11.76)^a^	2.1 (0.98–11.2)
**High level 1**											
Egg intake											
1 egg/day	249	83 (33.3)	1.0 (0.65–1.52)	1.0 (0.69–1.64)	1.1 (0.73–1.79)	1.2 (0.62–1.53)	92 (36.9)	1.1 (0.72–1.66)	1.1 (0.73–1.73)	1.2 (0.79–1.94)	1.3 (0.75–1.92)
>1 egg/day	207	68 (32.9)	1.1 (0.70–1.73)	1.1 (0.74–1.89)	1.0 (0.62–1.65)	1 (0.65–1.54)	84 (40.6)	1.3 (0.87–2.10)	1.3 (0.86–2.26)	1.2 (0.79–2.03)	1.2 (0.75–2.10)
<1 egg/day	196	80 (40.8)	1.7 (1.06–2.71)^a^	1.6 (1.03–2.70)^a^	1.7 (1.05–2.81)^a^	1.6 (0.83–3.12)	74 (37.8)	1.5 (0.97–2.49)	1.3 (0.86–2.26)	1.3 (0.84–2.28)	1.2 (0.81–2.18)
**High level 2**											
Egg intake											
1 egg/day	167	62 (37.1)	1.3 (0.81–2.13)	1.4 (0.85–2.30)	1.4 (0.86–2.41)	1.6 (0.84–2.31)	63 (37.7)	1.3 (0.82–2.14)	1.3 (0.83–2.24)	1.4 (0.85–2.38)	1.3 (0.81–2.43)
>1 egg/day	157	49 (31.2)	1.2 (0.74–2.09)	1.2 (0.75–2.19)	1.4 (0.81–2.56)	1.8 (0.92–2.34)	73 (46.5)	1.8 (1.13–3.01)^a^	1.9 (1.17–3.20)^a^	2.2 (1.31–3.91)^a^	2.3 (1.28–4.01)^a^
<1 egg/day	132	45 (34.7)	1.9 (1.06–3.45)^a^	2.0 (1.11–3.70)^a^	1.9 (1.07–3.66)^a^	2.5 (1.10–3.82)^a^	66 (50.0)	2.7 (1.58–4.89)^a^	2.7 (1.51–4.84)^a^	2.7 (1.50–4.90)^a^	2.9 (1.54–4.96)^a^

Normal blood pressure was the reference; compared with the medium level PA and 1 egg/day group, ^a^P < 0.05; Model 1 adjusted gender, age, occupation and personal annual income; model 2 adjusted smoking, drinking, cereal and tuber, livestock and poultry meat and milk based on model 1; model 3 adjusted BMI and family history of CVD based on model 2.

## Discussion

This study found that compared with the medium level PA group, the association of the high level 1 PA group with hypertension was similar to the medium level PA group, suggesting that moderate high level PA was associated with the low examination rate of hypertension, similar to previous studies^13^. A large number of studies have shown that moderate and high level PA can reduce the risk of hypertension^14,15^. Possible mechanisms include: PA can reduce sympathetic nerve activity or increase vagus nerve tension, thereby reducing peripheral resistance and lowering blood pressure. In addition, PA can reduce norepinephrine levels by about 30%, which may be parallel to blood pressure reduction^14^. Therefore, the hypertension guidelines recommend PA as a lifestyle behavior to prevent hypertension^13,15^. This study also found that compared with the medium level PA group, the examined abnormal rate in the high level 2 PA group was increased, OR was 1.8. Studies have confirmed that when exercise intensity is too high, cardiac myocytes can produce a lot of free radicals, mitochondrial overload, and suffer from ischemic and hypoxic injury, which brings a certain degree of damage to the heart, increasing the risk of hypertension^16^. Besides, the self-reported rate in the high level 2 PA group was increased than the medium level PA group in model 3, OR was 1.5. Maybe hypertension impels patients to do PA. Previous studies have shown that low level PA increased the risk of hypertension^6,14^, this study found that self-reported rate and examined abnormal rate in the low level PA group all increased compared with the medium level PA group, but all the difference was no statistically significant, that may be caused by the small sample size of the low level PA group, which also was the limitation of this study.

The results of this study showed that compared with the 1 egg/day group, the self-reported rate and examined abnormal rate were all increased in the <1 egg/day group, and OR were 1.4 and 1.4 in model 3, respectively, which was similar to the previous study results^1^. A recent prospective study reported that compared with non-consumers, daily egg consumption (usual amount 0.76 egg/day) was associated with lower risk of CVD (OR 0.89)^1^. Although dietary cholesterol in eggs increased serum total cholesterol and low density lipoprotein (LDL), it also increased high density lipoprotein (HDL)^1,17^. In addition, other ingredients in eggs such as phospholipids, high-quality proteins and carotenoids may contribute to cardiovascular health^1^. In this study, the self-reported rate decreased and examined abnormal rate increased in the >1 egg/day group compared with the 1 egg/day group, but all the difference was not statistically significant. For patients with hypertension, the focus on healthy diet increased due to the decline of physical fitness, so that they supplemented eggs with reasonable. But these patients may be too few to make a difference for the self-reported rate. The average of egg intake of >1 egg/day group was 135 ± 5.34 g/day in this study, the equivalent of 2.7 eggs/day, far more than the Chinese Nutrition Society recommend for adults (40 ~ 50 g/day). Excess egg supplement may make the increase of negative effect of serum total cholesterol and LDL more than the increase of positive effect of HDL and phospholipids on hypertension, which resulted in the increase of the examined abnormal rate. But compared with 1 egg/day, 2.7 eggs/day may be not enough to make a statistically significant increase in the examined abnormal rate.

This study found that compared with the medium level PA and 1 egg/day group, the increase of self-reported rate and examined abnormal rate in the high level 2 PA and <1 egg/day group were all significant, OR was 2.5 and 2.9 in the model 3, respectively, which suggested no egg supplement during increasing PA was associated with hypertension. This may be related to high-quality protein supplement of egg during exercise. Concordant with this finding, Figueroa *et al*.^11^ showed that combining exercise with milk can reduce systolic blood pressure. Williams^18^ came out that vegetarian athletes had lower HDL than omnivores, which may be associated with lack of animal and plant protein. However, there were few studies on interaction associations of PA and egg intake with hypertension in China. Feng *et al*.^5^ found that the risk of hypertension was significantly reduced when the combination of dietary pattern (dominated by high-protein foods) and PA, which was consistent with this study. Numerous studies have shown that long and high intense exercise required protein supplements^19,20^. Xie *et al*.^19^ showed that the metabolism of the body increased during strenuous exercise. Wang *et al*.^20^ found that in long-distance running, lack of protein supplements not only affect the effect of physical exercise, but also can result in sports anemia, which may cause harm to athletes’ health. According to the Chinese Academy of Medical Sciences^21^, the protein requirements for medium, heavy and very heavy manual workers were 1.2, 1.4 and 1.6 g/kg body weight, respectively.

However, too much protein during excessive high level PA is also bad for your health^19,20^. This study showed that compared with the medium level PA and 1 egg/day group, examined abnormal rate in the high level 2 PA and >1 egg/day group had significant increase, OR was 2.3 in model 3, which was consistent with the results of previous studies^19,20^. Xie *et al*.^19^ showed that the total protein intake of athletes should account for 13–15% of the total energy, and excessive intake will cause other metabolic abnormalities. Wang *et al*.^20^ found that eating too much protein during PA can have adverse consequences, excess protein carries the fat that causes vascular sclerosis, including cholesterol and these substances that can cause cardiovascular diseases. But in this study, we have not found significant increase of self-reported rate in the high level 2 PA and >1 egg/day group. The possible reason is that the self-reported hypertension group consciously control the amount of PA due to advised by doctors, which result in the median of PA of high level 2 PA and >1 egg/day group in self-reported hypertension population (6606 METs) lower than that in examined abnormal blood pressure population (7066 METs).

There are some limitations in our study. First, the assessment of PA and egg intake are self-reported and thus is imprecise. Second, the sample size of the low level PA group is too small to examine the association of low level PA with hypertension. Third, results of this study are limited to middle-aged and older people and not for young people. Finally, this is cross-sectional study and thus the association of PA and egg intake with hypertension should be further examined in large cohort studies.

## Conclusion

To sum up, excessive high level PA and inadequate egg intake were associated with hypertension, respectively, and the association further increased when both existed at the same time. Besides, moderate PA combined with reasonable egg intake was associated with the low examination rate of hypertension.

## Methods

### Study population

This study was a cross-sectional study of 2189 participants (aged ≥50 years and have resided in the Mentougou for 6 months or more in the past 12 months) in the Mentougou district of Beijing, China. Participants were selected by a three-stage stratified random sampling performed on 9 towns and 4 streets on the basis of the survey of Adult Chronic Diseases and Risk Factors Monitoring in the Mentougou from April 2016 to June 2017. In this study, face-to-face interviews were conducted by trained local interviewers using a questionnaire for demographic information, behavior and lifestyle information, and health status of participants; physical examination for height, weight and blood pressure; laboratory examination for blood routine and blood biochemical indexes. Moreover, we excluded participants whose PA data missing or the total daily accumulated time of all PA was over 960 min(16 h)^22^ for analyzing the PA distribution and the association between PA with hypertension, and the remaining was 2026; excluded participants whose egg intake data missing for analyzing the distribution of egg intake and the association between egg intake with hypertension, with the remaining 2136; excluded participants who was absence of PA (or the total daily cumulative time of all PA more than 960 min(16 h)) and egg intake data for analyzing the interaction association of PA and egg intake with hypertension, the rest were 1978.

### Ethical approval

Prior to the start of this study, it has been approved by the Biomedical Ethics Committee of Peking University, carried out in accordance with the principles of the Declaration of Helsinki, and all subjects have signed the informed consent.

### Assessment of PA, egg intake, and covariates

The questionnaire of PA in this study referred to long volume of the International Physical Activity Questionnaire (IPAQ) that was one of the most effective and widely used questionnaires on the PA level of adults. According to the grouping standard suggested by the working group on IPAQ^22^, individuals could be divided into the low level PA group, the medium level PA group and the high level PA (Table 4), and further individuals belonged to the high level PA group were subdivided into the high level 1 PA group (Q1 - Q3) and the high level 2 PA group (Q4 - Q5) by five points (Q1 - Q5) of the total metabolic equivalent.

**Table 4:** Categories criteria for level of PA.

Group	Standard
high level PA	Meet any of the following 2 criteria:
	a) Vigorous-intensity activity on at least 3 days achieving a minimum total physical activity of at least 1500 MET-minutes/week
	b) 7 or more days of any combination of walking, moderate-intensity or vigorous-intensity activities achieving a minimum Total physical activity of at least 3000 MET-minutes/week
Medium level PA	Meet any of the following 3 criteria:
	a) 3 or more days of vigorous-intensity activity of at least 20 minutes per day
	b) 5 or more days of moderate-intensity activity and/or walking of at least 30 minutes per day
	c) 5 or more days of any combination of walking, moderate-intensity or vigorous intensity activities achieving a minimum of at least 600 MET-minutes/week
low level PA	Meet any of the following 2 criteria:
	a) No activity is reported
	b)Some activity is reported but not enough to meet categories above

The questions of egg intake in questionnaire contained the frequency (daily, per week, per month, and never or rarely) in the past 1 month; times in the frequency and the amount in each time. We calculated the daily amount of egg intake of individual during the past 1 month and then individuals were divided into <1 egg/day group, 1 egg/day group and >1 egg/day group referring to the dietary guidelines from the Chinese Nutrition Society recommend^23^.

In this study, we also collected covariates including demographic information (such as gender, age, occupation and income), other behavior and lifestyle information (such as smoking, drinking, the intake of cereal and tuber/livestock and poultry meat/milk), and health status (such as diabetes, dyslipidemia) of participants through a self-designed unified questionnaire. Height and weight were all collected through physical examination, and BMI was calculated from the weight in kilograms divided by the square of the height in meters (kg/m^2^).

### Definition of hypertension

Blood pressure was measured via electronic manometer and the participants were in sitting position. Each participant was examined three times of resting blood pressure in this study. All measurements and medical examinations were performed by trained health care workers. Diagnostic criteria of hypertension were systolic blood pressure (SBP) above 140 mmHg or/and diastolic blood pressure (DBP) above 90 mmHg^24^. For participants, they may have following situations: (1) self-reported diagnosis of hypertension by the community or above hospitals in the past; (2) self-reported use of an antihypertensive medication; (3) mean blood pressure was above 130/80 mm Hg but didn’t meet diagnostic criteria during this examination; (4) mean blood pressure was above 140/90 mm Hg in this examination. Self-reported hypertension was defined if individual met (1) or (2); prehypertension was defined if individual didn’t belong to self-reported hypertension and only met (3); hypertension incidence was defined if individual didn’t belong to self-reported hypertension and only met (4); normal was defined if individual didn’t meet any of above situations. Besides, examined abnormal blood pressure was defined, including prehypertension and hypertension incidence, because the associations of PA and egg intake with prehypertension were consistent with hypertension incidence in this study and the American Hypertension Guideline 2017^25,26^ also adjusted the diagnostic criteria to 130/80 mm Hg although Chinese criteria has not.

### Statistical analysis

Using SPSS 20.0 statistical software for data analysis. Number (ratio or percentages) was used to describe, and chi-square test was used to compare between groups for categorical data; continuous variables were described by mean (standard deviation, SD), and t test or variance analysis was used for inter-group comparison. Multiple logistic regression was performed to evaluate the independent and interaction associations of PA and egg intake with hypertension. Firstly, the main effects of PA and egg intake on hypertension were analyzed, respectively. And then the interactions effects of PA and egg intake with hypertension were investigated by construction of interaction terms PA × egg intake. Both the main effect and the interaction effect are adjusted by the covariate models. This study included 2 covariate models. Model 1 adjusted gender(men or women), age(≥65 or <65 years), occupation (productive labor, services, retired, and others) and personal annual income(≥2800, <2800 yuan/year). Model 2 adjusted smoking(≥1, <1 cigarette/day), drinking(≥1, <1time/month), cereal and tuber(<250, 250∼, 400∼g/day), livestock and poultry meat(<40, 40∼, 75∼g/day) and milk(<300, ≥300 ml/day) on the basis of model 1. Model 3 adjusted BMI(<24.0, 24.0∼, 28.0∼kg/m^2^) and family history of CVD(yes or no) based on model 2. The statistical significance was set at two-tailed P < 0.05.

## Data Availability

The datasets generated during and/or analyzed during the current study are available from the corresponding author on reasonable request.

## References

[1] Qin C (2018). Associations of egg consumption with cardiovascular disease in a cohort study of 0.5 million Chinese adults. Heart.

[2] Kearney PM (2005). Global burden of hypertension: analysis of worldwide data. Lancet.

[3] Ma L, Wu Y, Wang W, Chen W (2018). Interpretation of key points of Chinese Cardiovascular Disease Report 2017. Chinese Journal of Cardiovascular Medicine.

[4] National Health and Family Planning Commission. *Report on nutrition and chronic diseases of Chinese residents* (*2015*). (People’s Medical Publishing House, 2015).

[5] Feng H (2016). Combined effect of dietary mode and physical activity of urban residents in Nanjing on the incidence of hypertension. Chinese Public Health.

[6] Diaz KM (2017). Physical activity and incident hypertension in African Americans: the Jackson Heart Study. Hypertension.

[7] Huai P (2013). Physical activity and risk of hypertension: a meta-analysis of prospective cohort studies. Hypertension.

[8] Semlitsch T (2013). Increasing physical activity for the treatment of hypertension: a systematic review and meta-analysis. Sports Med.

[9] Alexander DD, Miller PE, Vargas AJ, Weed DL, Cohen SS (2016). Meta-analysis of egg consumption and risk of coronary heart disease and stroke. Journal of the American College of Nutrition.

[10] Liu CP (2006). Carbohydrate and protein supplement for badminton players. Shanxi sport science and technology.

[11] Figueroa A (2014). Effects of Milk Proteins and Combined Exercise Training on Aortic Hemodynamics and Arterial Stiffness in Young Obese Women With High Blood Pressure. American Journal of Hypertension.

[12] Pan YG (2005). Great potential of egg production. China poultry.

[13] Brook RD (2013). Beyond medications and diet: alternative approaches to lowering blood pressure: a scientific statement from the American Heart Association. Hypertension.

[14] Borjesson M (2016). Physical activity and exercise lower blood pressure in individuals with hypertension: narrative review of 27 RCTs. British Journal of Sports Medicine.

[15] Mancia G (2013). 2013 ESH/ESC guidelines for the management of arterial hypertension: the Task Force for the Management of Arterial Hypertension of the European Society of Hypertension (ESH) and of the European Society of Cardiology (ESC). European Heart Journal.

[16] Liu J (2015). Impact of physical activity on cardiovascular diseases in residents of Hubei province. Chinese Cardiovascular Research.

[17] Berger S (2015). Dietary cholesterol and cardiovascular disease: a systematic review and meta-analysis. American Journal of Clinical Nutrition.

[18] Williams TP (1997). Interactive effects of exercise, alcohol, and vegetarian diet on coronary artery disease risk factors in 9242 runners: the National Runners’ Health Study. The American Journal of Clinical Nutrition.

[19] Xie ZH (2006). Study on the relationship between protein intake and nitrogen balance in body of male juvenile road cyclists. Journal of Tianjin sport university.

[20] Wang H, Yue XP (2005). Effects of protein on improving athletic performance of athletes. Journal of sports research and education.

[21] Institute of Health & Chinese Academy of Medical Sciences. *Food Composition Table*. 369-370 (People’s Health Publishing House, 1976).

[22] Fan M, Lu J, He P (2014). Chinese guidelines for data processing and analysis concerning the International Physical Activity Questionnaire. *Chinese*. Journal of Epidemiology.

[23] Shi X (2016). Publication of Chinese dietary guidelines (2016). Chinese Maternal And Child Health Study.

[24] China Hypertension Control Guidelines Revision Committee (2011). Chinese Hypertension Prevention Guidelines. Chinese Journal of Hypertension.

[25] Sun N (2017). Interpretation of key points and our thoughts in the American hypertension guide 2017. Chinese Journal of Hypertension.

[26] Whelton PK (2018). 2017 ACC/AHA/AAPA/ABC/ACPM/ AGS/APhA/ASH/ASPC/NMA/PCNA Guideline for the prevention, detection, evaluation, and management of high blood pressure in adults. Journal of the American College of Cardiology.

